# An Update on Antioxidative Stress Therapy Research for Early Brain Injury After Subarachnoid Hemorrhage

**DOI:** 10.3389/fnagi.2021.772036

**Published:** 2021-12-06

**Authors:** Fa Lin, Runting Li, Wen-Jun Tu, Yu Chen, Ke Wang, Xiaolin Chen, Jizong Zhao

**Affiliations:** ^1^Department of Neurosurgery, Beijing Tiantan Hospital, Capital Medical University, Beijing, China; ^2^China National Clinical Research Center for Neurological Diseases, Beijing, China; ^3^Center of Stroke, Beijing Institute for Brain Disorders, Beijing, China; ^4^Beijing Key Laboratory of Translational Medicine for Cerebrovascular Disease, Beijing, China; ^5^The General Office of Stroke Prevention Project Committee, National Health Commission of the People’s Republic of China, Beijing, China; ^6^Institute of Radiation Medicine, Chinese Academy of Medical Sciences, Peking Union Medical College, Tianjin, China; ^7^Savaid Medical School, University of Chinese Academy of Sciences, Beijing, China

**Keywords:** oxidative stress, subarachnoid hemorrhage, early brain injury, delayed cerebral ischemia, experimental – animal models

## Abstract

The main reasons for disability and death in aneurysmal subarachnoid hemorrhage (aSAH) may be early brain injury (EBI) and delayed cerebral ischemia (DCI). Despite studies reporting and progressing when DCI is well-treated clinically, the prognosis is not well-improved. According to the present situation, we regard EBI as the main target of future studies, and one of the key phenotype-oxidative stresses may be called for attention in EBI after laboratory subarachnoid hemorrhage (SAH). We summarized the research progress and updated the literature that has been published about the relationship between experimental and clinical SAH-induced EBI and oxidative stress (OS) in PubMed from January 2016 to June 2021. Many signaling pathways are related to the mechanism of OS in EBI after SAH. Several antioxidative stress drugs were studied and showed a protective response against EBI after SAH. The systematical study of antioxidative stress in EBI after laboratory and clinical SAH may supply us with new therapies about SAH.

## Introduction

Aneurysmal subarachnoid hemorrhage (aSAH) is a devastating disease, mainly induced by the rupture of an intracranial aneurysm and linked to high levels of morbidity and mortality (Bor et al., 2008; Connolly et al., 2012; Macdonald and Schweizer, 2017; Chao et al., 2021). Although we have progressed in treatment, 40% of aSAH survivors remain dependent as a consequence of neurological disability and behavioral and cognitive impairments (Brathwaite and Macdonald, 2014; Etminan and Macdonald, 2017). Clinical studies have shown that cerebral vasospasm (CVS) is not the single contributor to delayed cerebral ischemia (DCI) and poor prognosis in patients with aSAH (Naraoka et al., 2018; Mayer et al., 2019; Schupper et al., 2020; Post et al., 2021; Takeuchi et al., 2021). At present, amassing laboratory evidence demonstrates that early brain injury (EBI), which happens within 72 h after aSAH, causes subsequently pathophysiological changes and poor outcomes (Kusaka et al., 2004). The pathological changes and mechanisms of EBI collectively contain increased intracranial pressure (ICP), oxidative stress (OS), neuroinflammation, blood–brain barrier (BBB) disruption, brain edema, and cell death. Among them, the OS responses include a wide variety of active and inactive substances, which play a substantial role in EBI after aSAH and may be associated with DCI and long-term outcomes (Cahill et al., 2006; Rowland et al., 2012; Sehba et al., 2012; Shao et al., 2020). Therefore, we should pay more attention to strategies targeting cerebral redox responses to some extent. In this review, we update the impact of OS in the occurrence and development of SAH and several major oxidative pathways and biomarkers related to EBI after SAH. Additionally, we also take an overlook for the potential therapeutic drugs.

Although widely accepted than other pathogenic mechanisms, DCI has not reached further improvement clinically. By the same token, the failure of clazosentan, despite mitigating moderate and severe vasospasm, has shifted the focus of preclinical and clinical research from DCI to EBI toward a more multifactorial etiology in recent times (Macdonald et al., 2008, 2011; Cahill and Zhang, 2009; Caner et al., 2012).

## Mechanisms of Early Brain Injury

The topic raised in 2004, EBI is a designation that refers to the damage occurring to the brain in the first 72 h ensuing the initial aneurysmal bleeding and preceding vasospasm, including the primary injury and its direct consequences (Kusaka et al., 2004). The mechanisms resulting in EBI after aSAH are multifactorial and complicated. Conventionally, they are partitioned into the following parts (Rowland et al., 2012): (1) mechanical: acute or chronic hydrocephalus (Asada et al., 2021; Toyota et al., 2021); (2) physiological: raised ICP, decreased cerebral perfusion pressure (CPP) and cerebral blood flow (CBF), impaired cerebral autoregulation (CA), vasoconstriction, global ischemia, and delayed edema; (3) ionic: impaired ionic homeostasis, Ca^2++^ overload, K^+^ efflux, and cortical spreading depression (CSD); (4) inflammatory: NO (nitric oxide)/NOS (nitric oxide synthase) pathway activation, endothelin-1 (ET-1) release, platelet activation; and (5) cell death: endothelial cells, neurons, and astrocytes.

Aside from the aforementioned classical mechanisms, atypical mechanisms are newly proposed. Diverse factors, such as micro-spasm rather than macro-spasm (Fumoto et al., 2019), microthrombosis (Fumoto et al., 2019), early cortical infarction (Hartings et al., 2017; Eriksen et al., 2019), white matter injury (WMI) (Wu Y. et al., 2017; Pang et al., 2019; Peng et al., 2019; Ru et al., 2021), endoplasmic reticulum (ER) stress (Xie et al., 2019; Xu et al., 2019; Xiong et al., 2020), and immune inflammation (Ju et al., 2020; Rubio-Perez et al., 2021; Zeyu et al., 2021), are involved in cell death-relevant mechanisms in EBI after aSAH. Admittedly, various mechanisms or contributors to early injury will consequently result in cell death of the catastrophic ictus. Nowadays, the cell death processes in study include apoptosis, necrosis, autophagy (Dou et al., 2017; Sun C. et al., 2019; Sun C. M. et al., 2019), necroptosis (Kooijman et al., 2014; Chen et al., 2017, 2018, 2019; Xie et al., 2017; Yang C. et al., 2018; Yuan et al., 2019; Fang et al., 2020; Xu H. et al., 2021), pyroptosis (Yuan et al., 2020; Xu P. et al., 2021; Zhang C. S. et al., 2021), and ferroptosis (Lee et al., 2010; Zhao et al., 2018; Fang et al., 2020; Sq et al., 2020; Cao et al., 2021; Li et al., 2021; Qu et al., 2021). All but the former two well-known cell death mechanisms remain novel types and cutting-edge hot topics. For example, autophagy is an important protective mechanism against apoptosis, and recombinant osteopontin (rOPN) inhibits neuronal apoptosis by activating autophagy and regulating autophagy-apoptosis interactions (Sun C. M. et al., 2019). Necroptosis, a caspase-independent mechanism, plays a crucial part in the pathophysiological process by reducing the number of abnormal cells in brain tissue. Recently, SAH-induced synaptic impairments mitigated by NEC-1 in the hippocampus that inhibits necroptosis in relation to the CREB-BDNF pathway were verified (Yang C. et al., 2018). Moreover, another type of cell death, SAH-induced neuronal pyroptosis, is ameliorated in part by postconditioning with hydrogen gas through the mitoK_ATP_/ERK1/2/p38 MAPK signaling pathway (Zhang C. S. et al., 2021). Unlike other types of cell death, such as necrosis and apoptosis, ferroptosis is a regulated process caused by an imbalance of the redox system. Cell death results in damaged structure and function of vessels and nerves, causing ultimately post-SAH dysfunction.

Typically, the elementary changes after SAH can be segmented into two periods: EBI and DCI. Pathological changes occurring in the initial stage of hemorrhage propagate and lead to inflammation, OS, and apoptosis. Studies showed that OS plays a key role in the pathogenesis of EBI after SAH.

## Oxidative Stress in Subarachnoid Hemorrhage

Although progress is made in cell death, the actual pathogenesis of EBI after SAH is still rarely understood. Several pieces of research demonstrate that OS is one of the basic drivers of EBI (Zhang et al., 2016c; Fumoto et al., 2019).

Relying on the activity of the producer and removal systems, OS has its yin and yang faces, but those covered in this review are the result of the dysregulation of reduction-oxidation (redox) reactions. Sies and Jones (2020) also defined the elevated constitution of various reactive oxygen species (ROS) resulting in all classes of molecular damage as “oxidative distress.” OS, a relative extra of ROS compared with antioxidants, has been associated with cardiovascular, neurodegenerative, and many other diseases. ROS is an overall term for derivatives of dioxygen not chemically precise, which serve as a normal character of aerobic life. Hydrogen peroxide (H_2_O_2_) and the O_2_^•–^ are pivotal agents produced by greater than 40 enzymes (Murphy, 2009; Go et al., 2015). Additionally, many other reactive categories are contained in redox signaling, for example, hydrogen sulfide and oxidized lipids (Fujii et al., 2010; Yu et al., 2014; Jarocka-Karpowicz et al., 2020).

After SAH, exhibiting aberrant redox hemostasis, the production of oxidants mainly comes from the disruption of mitochondria (Yan et al., 2015; Fan et al., 2021; Xu W. et al., 2021), extravascular hemolyzed blood (Vecchione et al., 2009; Deng et al., 2018), and enzymatic sources of free radicals (Sies et al., 2017; Yang et al., 2017; Sies and Jones, 2020). Intrinsic antioxidant activity can be exhausted by excessive free radicals, resulting in lipid peroxidation, protein breakdown, and DNA damage. Mention must be made that beyond the biology of H_2_O_2_ and O_2_^•–^, a significant area of ROS research is lipid-derived ROS. Oxidative DNA damage has also been widely distinguished by mutagenesis, DNA methylation, and chromatin structure. Although substantiation is also gathered about oxidative damage to RNA, the underlying functional effect has not yet been fully illustrated.

## Oxidative Stress in Early Brain Injury

For decades, the treatment of CVS and DCI has always been the focus of clinical practice. However, mounting evidence showed that even angiographic vasospasm is reversed, and clinical outcomes remain frustrating (Macdonald et al., 2008; Gomis et al., 2010). So far, nimodipine remains the only medication proven to lessen DCI and unfavorable outcomes. Therefore, new therapeutic regimens are promising (Hänggi et al., 2015).

**FIGURE 1:**
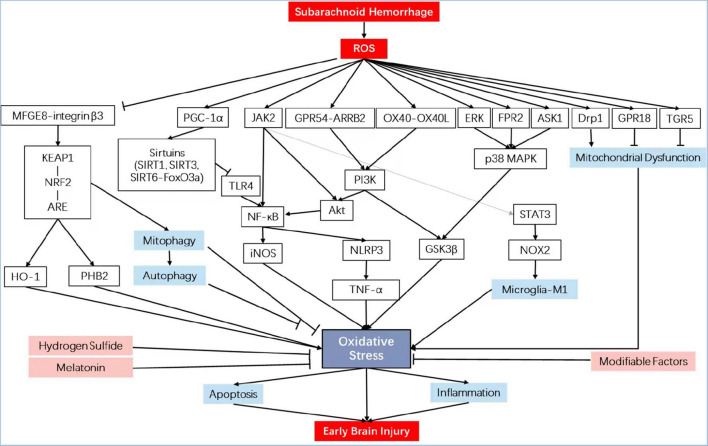
Schematic diagram illustrating the signaling pathways involved in oxidative stress in early brain injury. iNOS, inducible nitric oxide synthase; PHB2, prohibitin 2; NRF2, nuclear factor erythroid 2-related factor 2; HO-1, heme oxygenase-1; NF-κB, nuclear factor kappa-B; KEAP1, Kelch-like epichlorohydrin-associated protein 1; ARE, antioxidant response element; MFGE8, milk fat globule–EGF factor-8; TLR4, toll-like receptor 4; TNF-α, tumor necrosis factor-α; GPR18, G protein-coupled receptor 18; p38 MAPK, mitogen-activated protein kinase; FPR2, formyl peptide receptor 2; ASK1, apoptosis signal-regulating kinase 1; Drp1, dynamin-related protein 1; OX40L, OX40 cognate ligand-protein; GPR54, G protein-coupled receptor 54; ARRB2, β-arrestin 2; GSK3β, glycogen synthase kinase-3β; PI3K, phosphatidylinositol 3-kinase; TGR5, trans-membrane G protein-coupled receptor-5; PGC-1α, peroxisome proliferators-activated receptor-γ coactivator-1α; NLRP3, NLR family, pyrin domain containing 3.

Previously, the aspecific erasure of ROS using antioxidant compounds was unsuccessful in offsetting SAH initiation. However, regulating specific ROS-mediated signaling pathways offers a viewpoint, mainly containing enzymatic defense systems like those regulated by the nuclear factor erythroid 2 (NF-E2)-related factor 2 (Nrf2) (Zolnourian et al., 2019; Sies and Jones, 2020) and PI3K/Akt, the role of key molecules such as melatonin, sirtuins, and hydrogen sulfide, and the modifiable factors that are corporately thought as the exposome [by way of illustration, nutrition (Liu et al., 2015), lifestyle, and irradiation] (see overview in Table 1 and Figure 1). Discovering strategies for effectively detoxifying free radicals has become a theme of great interest from both practical and academic viewpoints.

**TABLE 1:** Clinical and experimental studies overview of OS in EBI after SAH.

	**Method/animal**	**Numbers (all/groups)**	**Drug/agent**	**Pathway**	**Effect**	**References**
KEAP1-NRF2-ARE pathway	Injection/rat	30/2	–	Nrf2-ARE	Nrf2 expression is upregulated in the cerebral artery of rats after experimental SAH	Wang et al., 2010
	Injection/rat	72/4	Sulforaphane	Nrf2-ARE	Nrf2-ARE pathway is activated in the brain after SAH, playing a beneficial role in EBI development, possibly through inhibiting cerebral oxidative stress by inducing antioxidant and detoxifying enzymes	Chen et al., 2011
	Perforation/rat	163/5	MitoQ/ML385	Keap1/Nrf2/PHB2	MitoQ inhibited oxidative stress related neuronal death by activating mitophagy via Keap1/Nrf2/PHB2 pathway	Zhang T. et al., 2019
	Injection/rat	60/5	RTA 408	Nrf2 and NF-κB	RTA 408 attenuated SAH-induced vasospasm through its reversal of SAH-induced changes in Nrf2, NF-κB, and iNOS	Tsai et al., 2020
	Injection/rabbit Perforation/rabbit	40/6	Tetramethyl- pyrazine nitrone (TBN)	Nrf2/HO-1	TBN ameliorated SAH-induced cerebral vasospasm and neuronal damage, attributed to its anti-oxidative stress effect and upregulation of Nrf2/HO-1	Wu et al., 2019
	Injection/rat	150/5	Aloperine (ALO)	Nrf2-ARE	ALO can ameliorate oxidative damage against EBI following SAH, most likely via the Nrf2-ARE survival pathway	Song et al., 2018
	Perforation/rat	210/4	Recombinant MFGE8	Integrinβ3/Nrf2/HO	Recombinant MFGE8 attenuated oxidative stress that may be mediated by integrin β3/nuclear factor erythroid 2–related factor 2/HO pathway after SAH	Liu et al., 2014
	Perforation/rat	221/4	TSG-6	NF-κB and HO-1	TSG-6 attenuated oxidative stress and apoptosis in EBI after SAH partly by inhibiting NF-κB and activating HO-1 pathway in brain tissue	Li et al., 2020
	Perforation/rat	96/4	Ursolic acid	TLR4/NF-κB	UA alleviated EBI by its anti-inflammatory properties, and the therapeutic benefit of post-SAH UA administration is due to its effect on inhibiting the activation of the TLR4/NF-κB signaling pathway	Zhang et al., 2014a
	Perforation/rat	132/3	Gastrodin	Nrf2/HO-1	The administration of gastrodin provides neuroprotection against early brain injury after experimental SAH	Wang et al., 2019
	Injection/rat	160/4	tert-Butylhy- droquinone (tBHQ)	Keap1/Nrf2/ARE	The administration of tBHQ abated the development of EBI and cognitive dysfunction in this SAH model for activation of the Keap1/Nrf2/ARE pathway	Wang et al., 2014
Mitochondrial pathway	Perforation/rat	76/4	–	–	Enhanced autophagy plays a protective role in early brain injury after SAH	Jing et al., 2012
	Perforation/rat	93/5	TT01001	–	mitoNEET activation with TT01001 reduced oxidative stress injury and neuronal apoptosis by improving mitochondrial dysfunction in EBI after SAH	Shi et al., 2020
	Perforation/rat	132/5	Docosahexaenoic acid	–	Prevent oxidative stress-based apoptosis after SAH, further improve mitochondrial dynamics-related signaling pathways	Zhang T. et al., 2018
	Perforation/rat	135/8	Resolvin D2	RvD2/GPR18	Upregulating GPR18 by RvD2 may improve neurological functions in different brain regions via multiple mechanisms	Zhang T. et al., 2021
	Perforation/rat	238/4	Lipoxin A4 (LXA4)	FPR2/p38	Exogenous LXA4 inhibited inflammation by activating FPR2 and inhibiting p38 after SAH	Guo et al., 2016
	Perforation/rat	32/4	Naringin	MAPK	Reduced the oxidant damage and apoptosis by inhibiting the activation of MAPK signaling pathway	Han et al., 2017a
	Injection/rat	232/4	Peroxiredoxin 1/2	ASK1/p38	Early expression of Prx1/2 may protect the brain from oxidative damage after SAH and may provide a novel target for treating SAH	Lu et al., 2019
	Perforation/rat	275/3	Mdivi-1	PERK/eIF2α/CHOP	Inhibition of Drp1 by Mdivi-1 attenuated early brain injury after SAH probably via the suppression of inflammation-related blood–brain barrier disruption and endoplasmic reticulum stress-based apoptosis	Fan et al., 2017
	Injection/rat	192/4	SS31	Mitochondrial apoptotic	SS31 could alleviate EBI after SAH through its antioxidant property and ability in inhibiting neuronal apoptosis, likely by modulating the mitochondrial apoptotic pathway	Shen et al., 2020
Other Pathway	Perforation/rat	165/10	ReOX40	OX40-OX40L/PI3K/AKT	ReOX40 attenuates neuronal apoptosis through OX40-OX40L/PI3K/AKT pathway in EBI after SAH	Wu et al., 2020
	Perforation/rat	249/5	Aggf1	PI3K/Akt/NF-κB	Exogenous Aggf1 treatment attenuated neuroinflammation and BBB disruption, improved neurological deficits after SAH in rats, at least in part through the PI3K/Akt/NF-κB pathway	Zhu et al., 2018
	Perforation/rat	196/11	Kisspeptin-54 (KP54)	GPR54/ARRB2/AKT/GSK3β	Administration of KP54 attenuated oxidative stress, neuronal apoptosis and neurobehavioral impairments through GPR54/ARRB2/AKT/GSK3β signaling pathway after SAH in rat	Huang et al., 2021
	Perforation/mouse	168/4	Apolipoprotein E	JAK2/STAT3/NOX2	apoE and apoE-mimetic peptide have whole-brain protective effects that may reduce EBI after SAH via M1 microglial quiescence	Pang et al., 2018
	Injection/rat	32/4	SC79	Iron accumulation	Disrupted iron homeostasis could contribute to EBI and Akt activation may regulate iron metabolism to relieve iron toxicity, further protecting neurons from EBI after SAH	Hao et al., 2016
	Injection/rat	319/4	SC79	Akt/GSK3β	SC79 exerts its neuroprotective effect likely through the dual activities of anti-oxidation and antiapoptosis	Zhang et al., 2016a
	Perforation/rat	84/4	Scutellarin (SCU)	Erk5-KLF2-eNOS	SCU could attenuate vasospasm and neurological deficits via modulating the Erk5-KLF2-eNOS pathway after SAH	Li Q. et al., 2016
	Injection/rat	120/3	Purmorphamine (PUR)	Sonic hedgehog	PUR exerts neuroprotection against SAH-evoked injury in rats, mediated in part by antiapoptotic and antioxidant mechanism, upregulating phospho-ERK levels, mediating Shh signaling molecules in the PFC	Hu et al., 2016
	Perforation/rat	199/5	TGR5/INT-777	cAMP/PKCε/ALDH2	The activation of TGR5 with INT-777 attenuated oxidative stress and neuronal apoptosis via the cAMP/PKCε/ALDH2 signaling pathway	Zuo G. et al., 2019
	Perforation/rat	196/5	AVE 0991	Mas/PKA/p-CREB/UCP-2	Mas activation with AVE reduces oxidative stress injury and neuronal apoptosis through Mas/PKA/p-CREB/UCP-2 pathway after SAH	Mo et al., 2019
Melatonin	Injection/rabbit	48/4	Melatonin	–	Post-SAH melatonin administration may attenuate inflammatory response and oxidative stress in the spasmodic artery	Fang et al., 2009
	Human	169/2	Melatonin	–	Patients with higher serum melatonin concentrations are more likely to have a poor prognosis	Zhan et al., 2021
	Perforation/mouse	–/3	Melatonin	Sirt3/SOD2 and Bax/Bcl-2/CC3	Melatonin provided protection from the effects of EBI following SAH by regulating the expression of murine SIRT3	Yang S. et al., 2018
	Perforation/mouse	–/3	Melatonin	NRF2 and mitophagy	By increasing the expression of NRF2, the mitophagy induced by melatonin provided protection against brain injury post-SAH	Sun et al., 2018
	Injection/rat	72/4	Melatonin	Nrf2-ARE	Through activating Nrf2-ARE pathway and modulating cerebral oxidative stress by inducing antioxidant and detoxifying enzymes	Wang et al., 2012
	Injection/rat	80/4	Melatonin	TLR4	Post-SAH melatonin administration might be due to its salutary effect on modulating TLR4 signaling pathway	Dong et al., 2016
	Perforation/rat	56/3	Melatonin	Mitochondrial	The mechanism of these antiapoptosis effects was related to the enhancement of autophagy, which ameliorated cell apoptosis via a mitochondrial pathway	Chen et al., 2014b
	Perforation/rat	77/3	Melatonin	Tight junction and pro-inflammatory	Melatonin prevents disruption of tight junction proteins which might play a role in attenuating brain edema secondary to BBB dysfunctions by repressing the inflammatory response in EBI after SAH	Chen et al., 2014a
Sirtuins	Injection/rat	262/4	Activator 3	SIRT1	SIRT1 plays an important role in endogenous neuroprotection by deacetylation and subsequent inhibition of FoxOs-, NF-κ B-, and p53-induced oxidative, inflammatory and apoptotic pathways	Zhang et al., 2016
	Injection/rat Injection/mouse	422/8	Astaxanthin (ATX)	SIRT1/TLR4	ATX treatment inhibits TLR4-mediated inflammatory injury by increasing SIRT1 expression after SAH	Zhang X. et al., 2019
	Injection/rat	96/4	Astaxanthin (ATX)	Nrf2-ARE	ATX treatment alleviated EBI in SAH model, possibly through activating the Nrf2-ARE pathway by inducing antioxidant and detoxifying enzymes	Wu et al., 2014
	Injection/rat Injection/rabbit	325/8 20/4	Astaxanthin (ATX)	–	ATX administration could alleviate EBI after SAH, potentially through its powerful antioxidant property	Zhang et al., 2014
	Injection/rat Injection/mouse	213/5 –	Fucoxanthin (Fx)/EX527	Sirt1	Fx provided protection against SAH-induced oxidative insults by inducing Sirt1 signaling	Zhang X. S. et al., 2020
	Injection/rat Injection/mouse	159/6 57/2	Salvianolic acid B	SIRT1 and Nrf2	SalB provides protection against SAH-triggered oxidative damage by upregulating the Nrf2 antioxidant signaling pathway, which may be modulated by SIRT1 activation	Zhang X. et al., 2018
	Perforation/rat	68/4	Salvianolic acid A	ERK/P38/Nrf2	SalA also modulated Nrf2 signaling, and the phosphorylation of ERK and P38 MAPK signaling in SAH rats	Gu et al., 2017
	Injection/mouse	132/4	LV-shPGC-1a	PGC-1a/SIRT3	The detrimental PGC-1a/SIRT3 pathway, involving regulation of the endogenous antioxidant activity against neuronal damage	Zhang K. et al., 2020
	Perforation/rat	200/5	Bexarotene	PPARγ/SIRT6/FoxO3a	The anti-neuroinflammatory effect was at least partially through regulating PPARγ/SIRT6/FoxO3a pathway	Zuo Y. et al., 2019
Hydrogen sulfide	Injection/rat	96/4	Hydrogen sulfide	–	NaSH as an exogenous H_2_S donor could significantly reduce EBI induced by SAH	Cui et al., 2016
	Injection/rat	134/5	L-cysteine	CBS/H_2_S	L-cysteine may play a neuroprotective role in SAH by inhibiting cell apoptosis, upregulating CREB-BDNF expression, and promoting synaptic structure via the CBS/H_2_S pathway	Li et al., 2017
	Perforation/rat	35/3	Hydrogen gas	–	The first report demonstrating that high dose hydrogen gas therapy reduces mortality and improves outcome after SAH	Camara et al., 2019
	Perforation/rat	182/5	Hydrogen gas	ROS/NLRP3	Hydrogen inhalation can ameliorate oxidative stress related endothelial cells injury in the brain and improve neurobehavioral outcomes in rats following SAH related to the inhibition of activation of ROS/NLRP3 axis	Zhuang et al., 2019
	Injection/rabbit	72/4	Hydrogen-rich saline (HS)	–	Treatment with hydrogen in experimental SAH rabbits could alleviate brain injury via decreasing the oxidative stress injury and brain edema	Zhuang et al., 2012
	Perforation/rat	129/4	Hydrogen-rich saline (HS)	NF-κB	HS may inhibit inflammation in EBI and improve neurobehavioral outcome after SAH, partially via inactivation of NF-κB pathway and NLRP3 inflammasome	Shao et al., 2016
	Injection/rat	244/8	Sodium/hydrogen exchanger 1 (NHE1)	–	NHE1 participates in EBI induced by SAH through mediating inflammation, oxidative stress, behavioral and cognitive dysfunction, BBB injury, brain edema, and promoting neuronal degeneration and apoptosis	Song et al., 2019
	Perforation/mouse	–/5	CO	–	First report to demonstrate that CO minimizes delayed SAH-induced neurobehavioral deficits	Kamat et al., 2019
Modifiable factors	Injection/rat	120/5	Gp91ds-tat/GKT137831/apocynin	–	Nox4 should contribute to the pathological processes in SAH-induced EBI, and there was not an overlay effect of Nox2 inhibition and Nox4 inhibition on preventing SAH-induced EBI	Zhang L. et al., 2017
	Injection/rabbit	40/5	Telmisartan	Trx/TrxR	Downregulation of TXNIP and upregulation of Trx/TrxR	Erdi et al., 2016
	Injection/rat	24/3	Verapamil	Antioxidant and antiapoptotic	Intrathecal verapamil can prevent vasospasm, oxidative stress, and apoptosis after experimental subarachnoid hemorrhage	Akkaya et al., 2019
	Perforation/rat	21/3	3,4-dihydroxyphenylethanol (DOPET)	–	Free radical scavenging capacity	Zhong et al., 2016
	Perforation/rat	40/4	3,4-dihydroxyphenylethanol (DOPET)	Akt and NF-κB	DOPET attenuates apoptosis in a rat SAH model through modulating oxidative stress and Akt and NF-κB signaling pathways	Fu and Hu, 2016
	Perforation/rat	80/4	Propofol/LY294002	PI3K/Akt	Propofol attenuates SAH-induced EBI by inhibiting inflammatory reaction and oxidative stress, which might be associated with the activation of PI3K/Akt signaling pathway	Zhang H. B. et al., 2019
	Perforation/rat	248/10	Wnt-3a	Frz-1/aldolase C/PPAN	Intranasal administration of wnt-3a alleviates neuronal apoptosis through Frz-1/aldolase C/PPAN pathway in the EBI of SAH rats	Ruan et al., 2020
	Perforation/rat	48/3	Preconditioning exercise	Nrf2/HO-1 14–3-3γ/p-β-catenin Ser37/Bax/caspase-3	Preconditioning exercise ameliorates EBI after SAH	Otsuka et al., 2021

### KEAP1-NRF2-ARE Pathway

An important “sensors” protein that captures specific metabolic information after SAH and transforms it into an appropriate response is Kelch-like ECH-associated protein 1 (Keap1), which contains reactive cysteine residues that collectively respond to ROS resulting from heme. Covalent modification of Keap1 leads to reduce ubiquitination and accumulates Nrf2 (Bollong et al., 2018). It is combined with a given DNA site, the antioxidant response element, modulating the transcription of a series of antioxidant enzymes (Zolnourian et al., 2019). Lots of researches on stroke models have substantiated that Nrf2 levels lift soon after the stroke. It first appears within 3 h and peaks within 24 h post insult, with the peri-infarct area markedly increasing (Yang et al., 2009; Tanaka et al., 2011). The study indicated that Nrf2 expression is significantly activated in neurons, astrocytes, leukocytes, microglia, endothelin cells, smooth muscle cells, and adventitial cells after SAH-induced brain insult (Wang et al., 2010). Wang et al., found that the Nrf2-ARE pathway is upregulated in rat models with experimental SAH in the time course at 12, 24, and 48 h (Chen et al., 2011). Since then, lots of known activators of the Keap1-Nrf2-ARE pathway were overwhelmingly carried out in the study. Although controversial conclusions are sometimes achieved due to pleiotropic and primary mechanisms, more effective inducers with less crossing activation of other pathways are identified and reach favorable outcomes.

In addition to the agents summarized by former researchers, such as sulforaphane, astaxanthin, curcumin, lycopene, tetra-butyl hydroquinone, dimethyl fumarate, melatonin, and erythropoietin, there are also many other experimental studies conducted more recently. Efforts to target the Nrf2 signaling pathway therapeutically have largely focused on covalent small molecule agonists of Keap1. Mitoquinone (MitoQ), effective in the prevention of mitochondrial dysfunction, restrained OS-related neuronal death by stimulating mitophagy through Keap1/Nrf2/PHB2 pathway after SAH in rats (Zhang T. et al., 2019). In the endovascular perforation mice SAH model, MitoQ treatment reduced OS, both short term and long term. Another activator of Nrf2, RTA 408, a new second-generation semisynthetic oleanane triterpenoid, manifested an antioxidant and anti-inflammatory phenotype (Reisman et al., 2014). After administrated intraperitoneally, vasospasm was reversed by RTA 408 through growth in Nrf2 and reduction in NF-κB (Tsai et al., 2020).

Intriguingly, performing as a downstream molecule in the Keap1-Nrf2-ARE pathway, heme oxygenase-1 (HO-1), also known as HSP32, could be induced by upregulating expression of Nrf2 (Wang and Doré, 2007; Jiang et al., 2020). After SAH, vasospasm and lipid peroxidation can be weakened by HO-1 through the improvement of clearance. Post-hemorrhagic administration of Nrf2 activator, tetramethylpyrazine nitrone (Wu et al., 2019), aloperine (Song et al., 2018), milk fat globule-epidermal growth factor 8 (MFGE8) (Liu et al., 2014, 2015), tumor necrosis factor-alpha stimulated gene-6 (Li et al., 2020), ursolic acid (UA) (Zhang et al., 2014a,b; Ding et al., 2017), gastrodin (Wang et al., 2019), tert-Butylhydroquinone (Wang et al., 2014), promotes posttranscriptional augment of both Nrf2 and HO-1, attenuates OS, and then reduces early brain damage, including brain edema, BBB damage, and cognitive impairment following SAH in animal models. Moreover, an additional NRF family member, namely, Nrf1 (Qian et al., 2019), responsible for ROS detoxification, participates in an effective treatment to moderate SAH-elicited EBI. Researchers concluded that HSP22 played a part in neuroprotective effects by regulating TFAM/Nrf1-triggered mitochondrial biogenesis with positive feedback, further attenuating OS and EBI (Fan et al., 2021).

### Mitochondrial Pathways

Shortly after the induction of SAH, EBI triggers mitochondrial disorder, in which many signaling molecules communicate with each other to control OS (Prentice et al., 2015). Thus, another pivotal key in alleviating EBI is discovering new options to keep normal mitochondrial activity by attenuating OS. The mechanisms for ROS generated by mitochondria are under the consensus that the production of ROS is maximal when the ingredients of the electron transport chains (ETCs) are superlatively impaired (Murphy et al., 1999; Moro et al., 2005). Particularly interacting with autophagy and apoptosis, activation of autophagic pathways attenuates EBI after SAH in rats (Jing et al., 2012; Shi et al., 2020; Xu W. et al., 2021). Among the mounts of antioxidant agents, docosahexaenoic acid (DHA), the so-called omega-3 fatty acid, reduces OS through enhancing mitochondrial dynamics in EBI (Zhang T. et al., 2018). Concretely, DHA reduced the number of ROC-positive cells, improved cell viability, attenuated malondialdehyde levels, and superoxide dismutase (SOD) stress. Furthermore, a metabolite of DHA, namely, resolvin D2 (RvD2), helps to defend EBI, especially in the cortex and hypothalamus (Zhang T. et al., 2021). The p38 mitogen-activated protein kinase (MAPK) is a major player in mitochondrial dysfunction after SAH (Sasaki et al., 2004; Yatsushige et al., 2007; Guo et al., 2016; Han et al., 2017a; Tomar et al., 2017; Lu et al., 2019). Recently, a p38 inhibitor, DJ-1, protects mitochondrial dysfunction by induction of translocation (Huang et al., 2018). Another selective inhibitor of Drp1, Mdivi-1, exerts neuroprotective effects against mitochondrial fission and OS (Fan et al., 2017; Wu P. et al., 2017). More recently, SS31 cell-membrane permeating mitochondria has been shown to exert potential neuroprotective effects (Petri et al., 2006). Via suppressing Bax translocation and cytochrome c release, SS31 ameliorated OS by inhibiting the mitochondrial pathway (Shen et al., 2020).

### Other Pathways

#### PI3K/Akt Pathway

The PI3K/Akt pathway is one of the important pathways that inhibit cell apoptosis and, therefore, plays a protective role against SAH. In recent years, antiapoptosis agents targeting this pathway generated the cross-talk with antioxidative effect (Wu et al., 2020). For example, Aggf1, also known as an angiogenic factor with G, patch, and FHA domain 1, in a recombinant human form, reduces BBB disruption and neuroinflammation through PI3K/Akt/NF-κB pathway after SAH in rats by significantly decreasing the level of myeloperoxidase (Zhu et al., 2018). For the first time, John H. Zhang et al., found that KISS1 siRNA knockdown (KD) aggravated neurological deficits and the brain expression of markers for OS in rats both 24 h and 28 days after SAH, suggesting that KP54 attenuated OS through activating GPR54/ARRB2/AKT/GSK3β pathway after SAH in rats (Mead et al., 2007; Huang et al., 2021). Another frontier hotspot involved in the PI3K/Akt pathway is the microglial polarization-mediated WMI (Xue et al., 2021). Additionally proposed by the John H. Zhang research group, low-density lipoprotein receptor-related protein-1 (LRP1), a scavenger receptor of apolipoprotein E (apoE), is validated for microglia polarization toward pro-antioxidative M2 phenotypes via Shc1/PI3K/Akt pathway after SAH in rats (Wu Y. et al., 2017; Rojo et al., 2018; Peng et al., 2019). Uniformly, apoE and apoE-mimetic peptides possess whole-brain protective effects that may reduce EBI after mice SAH via M1 microglial quiescence through the attenuation of the JAK2/STAT3/NOX2 signaling pathway axis (Pang et al., 2018). A shred of direct evidence presented by Kuanyu Li and his colleagues is that pAkt effectively inhibits iron accumulation, defense against OS, and ameliorates EBI in a model of experimental SAH in the temporal lobe (Jo et al., 2012; Hao et al., 2016; Zhang et al., 2016a). Interestingly, SC79 is an absorptive permeability without reported side effects, indicating a novel and promising delivery drug in patients with EBI after SAH.

#### More Recently Progressed Pathway

In addition to the Keap-Nrf2-ARE pathway and PI3K/Akt pathway, there are many other pathways that are oxidative related and proved to be effective. For instance, Scutellarin, a flavonoid from the Chinese herb *Erigeron breviscapus*, reduces vasospasm via the Erk5-KLF2-eNOS pathway after SAH (Li Q. et al., 2016). An agonist of the Shh co-receptor plays a part in neuroprotection against SAH-induced damage, mediated in part by antioxidant mechanisms, upregulating phospho-ERK levels, and mediating Shh signaling molecules in the prefrontal cortex (Hu et al., 2016). Benefitting from a broad distribution in neurons, astrocytes, and microglia, activation of TGR5 with INT-777 significantly attenuates OS through cAMP/PKCε/ALDH2 pathway after SAH in rats (Zuo G. et al., 2019). Recognized as a new component of the brain renin-angiotensin system, Mas is selectively target-activated by AVE 0991 and reduces OS through Mas/PKA/CREB/UCP-2 pathway (Mo et al., 2019). Moreover, 12/15-LOX is overexpressed in macrophages after SAH in mice, and restraint of the pathway attenuates brain injury and ameliorates unfavorable neurological outcomes. Progressing data support that various pathways may participate in the redox balance in EBI after SAH and needs to be added with a new insight of other underlying pathways.

### Melatonin

The number of data accumulated till now concerning the protective action of melatonin against OS is preponderant (Galano et al., 2011; Wu H. J. et al., 2017; Luo et al., 2019; Shao et al., 2020). Melatonin, a lipophilic amino acid that originated from tryptophan, N-acetyl-5-methoxytryptamine, is synthesized in the pineal gland and other organs and exhibits both direct and indirect antioxidant effects. Melatonin first reported the antioxidative function in preventing focal regions of injury via inducing HO-1 expression following a rat SAH model in 2002 (Martinez-Cruz et al., 2002). Before the post-clazosentan era, studies regarding the EBI experiments were still broadly focused on delayed brain injury, such as setting the assessment point at day 5 (Fang et al., 2009). Although melatonin shows no improvement in neurologic scores, the phenomenon is settled by large doses with immensely lessened mortality (Ayer et al., 2008a,b). However, the latest study shows that patients with higher serum melatonin concentrations are more likely to have a poor prognosis (Zhan et al., 2021). The increased concentrations of serum melatonin correlate with admission WFNS scores and mFS and serum melatonin appears as an independent predictor for poor 6-month prognosis after aSAH, with a high discriminatory ability for the risk of the poor outcome under the ROC curve, indicating that serum melatonin might serve as a promising prognostic biomarker for aSAH (Zhan et al., 2021). *In vivo* experiments exhibit that melatonin supplied protection from the effects of EBI after SAH by adjusting the expression of murine SIRT3 (one of the members of the sirtuin family) (Yang S. et al., 2018). Another two recent studies also denoted that melatonin plays a neuroprotective role by increasing the expression of NRF2-mitophagy and ER stress via inducing antioxidant, LC3-II/LC3-I, and Atg 5-mediated autophagy, NLRP3 inflammasome-mediated anti-inflammatory effects, and detoxifying enzymes post-SAH (Wang et al., 2012; Dong et al., 2016; Wu H. J. et al., 2017; Sun et al., 2018). Melatonin may reduce neurobehavioral dysfunction in the SAH model through the TRL4 pathway (Wang et al., 2013). Furthermore, melatonin reduces the EBI by influencing NLRP3 inflammasome-associated apoptosis (Dong et al., 2016) and inhibiting NF-κB activation and attenuating HO-1, NQO-1, and c-GCLC expressions (Jumnongprakhon et al., 2015). The mechanism of these antiapoptosis effects was linked to the improvement of autophagy through a mitochondrial pathway (Chen et al., 2014b). Melatonin inhibits the disruption of tight junction proteins possibly linked to the adjustment of proinflammatory cytokines (Chen et al., 2014a). Taken together, these results demonstrate that regulation of melatonin attenuates symptomatic dysfunction (Chen et al., 2015).

### Sirtuins

Sirtuins (SIRTs), including the seven SIRTs identified, are a family of deacetylases with homology. Lines of studies showed that SIRTs could modulate diverse biological functions, Sirtuin 1 (SIRT1) with antioxidative properties particularly. Demonstrating that sequential inhibition of forkhead transcription factors of the O class-, NF-κ B-, and p53-induced oxidative pathways, SIRT1 enhanced the neuroprotective role against EBI in rats (Zhang et al., 2016). A well-recognized antioxidant, astaxanthin, mitigates SAH-induced EBI by increasing SIRT1 and suppressing the TLR4 signaling pathway (Wu et al., 2014; Zhang et al., 2014; Zhang X. et al., 2019). Interestingly, derived from seaweeds, fucoxanthin (Fx) mitigates SAH-induced oxidative damage via the SIRT1-dependent pathway (Zhang X. S. et al., 2020). The activation of melanocortin 1 receptor with BMS-470539 immensely reduced EBI after SAH by restraining OS, apoptosis, and mitochondrial fission via the AMPK/SIRT1/PGC-1α signaling pathway (Xu W. et al., 2021). Modulated by SIRT1 activation, salvianolic acid B protects against SAH-triggered oxidative damage by upregulating the Nrf2 antioxidant signaling pathway (Zhang X. et al., 2018). Salvianolic acid homolog A also presented antioxidative, antiapoptotic, and anti-inflammatory properties (Gu et al., 2017; Zhang X. et al., 2018).

Other members of the SIRT family are increasingly studied recently. SIRT3, a type of NAD-dependent deacetylase, remarkedly activated *in vivo* and *in vitro* following SAH, is involved in the PGC-1a/SIRT3 pathway attenuating OS (Zhang K. et al., 2020). Drawing on the successful experience of the SIRT6 protective role of the heart from I/R injury via upregulating antioxidants and suppressing OS, the activation of RXR ameliorated neurological deficits after SAH at least partially via adjusting the PPARγ/SIRT6/FoxO3a pathway (Zuo Y. et al., 2019).

### Hydrogen Sulfide

Hydrogen sulfide (H_2_S), a neuromodulator, which can be generated in the CNS from L-cysteine by cystathionine-β-synthase (CBS), may prove protective effects in experimental SAH (Xiong et al., 2020). The hypothesis that signaling through hydrogen sulfide may mediate protection from DCI clinically in patients with SAH was proposed in 2011 (Grobelny et al., 2011) and further demonstrated by Yu et al. (2014). Afterward, hydrogen sulfide attenuated brain edema formation and promoted the secretion of inflammatory cytokines (Cui et al., 2016). Soon after the proinflammation demonstrated in EBI after SAH, exogenous hydrogen sulfide functioning as an antioxidant and antiapoptotic mediator, donated by NaSH and L-cysteine, could significantly reduce EBI (Cui et al., 2016; Li et al., 2017; Xiong et al., 2020). Inspired by inhaled hydrogen gas markedly decreasing OS on ischemia/reperfusion injury and stroke in rats (Ohsawa et al., 2007), hydrogen gas therapy was conducted, and the rate of survival and neurological deficits were improved in a pilot study as expected (Camara et al., 2019). Mechanistically, the above advantageous effects might be linked to the suppression of the ROS/NLRP3 axis (Zhuang et al., 2019). Similarly, hydrogen-rich saline exhibited the satisfying outcome of alleviating EBI through alleviating OS following experimental SAH in both rabbit and rat models (Zhuang et al., 2012; Shao et al., 2016). Furthermore, sodium/hydrogen exchanger 1 participates in EBI activated by SAH via mediating OS (Song et al., 2019). As abovementioned earlier, a recent study shows that postconditioning with hydrogen gas ameliorated SAH-induced neuronal pyroptosis (Zhang C. S. et al., 2021). Produced endogenously through HO, carbon oxide (CO) minimizes neurobehavioral deficits, indicating that posttreatment with CO gas or CO-donors can be further tested as a potential therapy against SAH (Kamat et al., 2019).

### Modifiable Factors

Peaking onset age between 50 and 60 years, many patients with aSAH have modifiable hypertension, dyslipidemia, diabetes mellitus, cardiovascular diseases, and so on (de Rooij et al., 2007; Zhang L. et al., 2017; Macdonald and Schweizer, 2017). To control the clinical status, they are recommended with antihypertensive drugs, statins, and so on, part of whom demonstrate the antioxidative effect in the laboratory, for example, telmisartan, ameliorates OS, and SAH-induced CVS (Erdi et al., 2016). One of the L-type calcium channel blockers, verapamil, can inhibit vasospasm, OS, and apoptosis following experimental SAH (Akkaya et al., 2019). Rosuvastatin, commonly used clinically, ameliorates EBI after SAH through restraining SOD formation and NF-κB activation in rats. Moreover, 3,4-dihydroxyphenylethanol may be a powerful agent in the treatment of EBI after SAH because of its free radical scavenging capacity and modulating the Akt and NF-κB signaling pathway (Zhong et al., 2016; Fu and Hu, 2016). In addition to the intervening abovementioned diseases, other drugs commonly used clinically, such as heparin (Hayman et al., 2017), albumin (Deng et al., 2021), and propofol (Zhang H. B. et al., 2019), all projected an antioxidative role. Some authors believe that preconditioning would provide the greatest chance of benefit but is obviously not effective (Mayor et al., 2013; Zolnourian et al., 2019; Ruan et al., 2020; Otsuka et al., 2021). Given the sophistication of brain damage after aSAH, therapeutic multimodality is promising. Supported by the evidence shown above, we suppose that the regular drug taken with high adherence may benefit favorable outcomes after aneurysm rupture than those who do not.

## Perspectives and Limitations

Shifted from phenotype research on OS in EBI to pathway-related research, the mechanisms become evident than at any time in the past (Zhang L. et al., 2017; Ye et al., 2018; Pan et al., 2021). However, since the term EBI was coined from an angle of preclinical mechanism insight, there is still a long way to apply clinically. Preclinical and clinical studies should proceed hand in hand, and none of the multi-omics and clinical trials should stop (Xu et al., 2017; Xu W. et al., 2018).

In addition to the traditional markers, such as malondialdehyde (MDA), SOD, reduced/oxidized glutathione (GSH/GSSG) ratio, and myeloperoxidase (MPO), new approaches that measure energetics and metabolomics of cells should be explored, such as the bioenergetic health index (BHI) (Chacko et al., 2016), to further guide the development of therapies. A lot of conducted studies showed that drugs introduced into the area of EBI after aSAH previously drew on the strength of the fields of ischemic stroke or traumatic brain injury. Furthermore, a close tracing of novel antioxidants is necessary, even in other disciplines with mutual adoption, promotion, and advancement, especially traditional Chinese medicine and nutrition (Wang et al., 2015; Zhang et al., 2015; Li Q. et al., 2016; Han et al., 2017b; Liu et al., 2017, 2020; Shao et al., 2019; Du et al., 2020; Wang T. et al., 2020). As the OS-related mechanisms and pathways have surfaced and matured, more modern technologies should emphasize their parts, such as designing a metabolite-derived protein alteration integrating glycolysis with Keap1-Nrf2 signaling directly (Bollong et al., 2018) or recombinant human drugs (Xie et al., 2018; Sun C. et al., 2019; Sun C. M. et al., 2019; Wang J. et al., 2020; Wu et al., 2020; Tu et al., 2021). Effective drugs validated in a laboratory should be carefully compared and put into clinical use properly. Typical drug pharmaceutical effects combined with evolving drug-loaded methods may be promising in future exploration. For example, the conventional antioxidant agent, curcumin, loaded into a nanosized PLGA-encapsulated the therapeutic potential was enhanced for precision medicine in downregulating the NF-κB pathway and preventing OS in EBI (Li X. et al., 2016; Zhang et al., 2016b; Cai et al., 2017; Zhang Z. Y. et al., 2017).

There are several limitations in this study. First, at present, there remains a translational cleft between experimental SAH and clinical SAH, especially aSAH. Although injection and endovascular perforation models are well established and invaluable, no model, even the *in vivo* cerebral aneurysmal models, could perfectly replicate the actual rupture of an aneurysm in human beings. Second, the unequivocal definition of EBI could not be circumvented in animal models with the variation assessment points in 3, 12, 24, or 72 h. Clear evaluation time is still up for debate. Third, based on a multifactorial pathophysiology after EBI, OS plays a pivotal part but is still only one of the considerable phenotypes with promising therapeutic strategies covering as many pathways as possible.

## Conclusion

Management of EBI after aSAH remains not only a challenge but also an opportunity. With in-depth understanding of oxidative pathophysiology of EBI, the way ahead becomes gradually clearer. Despite initial experimental studies demonstrating the effectiveness of the abovementioned antioxidants in EBI, these studies are comparatively rudimentary, with further translational medicine demanded to prove the utility of all of them clinically.

## Author Contributions

FL, RL, W-JT, KW, and YC contributed to the search and assessment of the available literature. FL and RL wrote the manuscript. XC and JZ helped to revise the manuscript to the final form. All authors contributed to the article and approved the submitted version.

## Conflict of Interest

The authors declare that the research was conducted in the absence of any commercial or financial relationships that could be construed as a potential conflict of interest.

## Publisher’s Note

All claims expressed in this article are solely those of the authors and do not necessarily represent those of their affiliated organizations, or those of the publisher, the editors and the reviewers. Any product that may be evaluated in this article, or claim that may be made by its manufacturer, is not guaranteed or endorsed by the publisher.
